# Multimodal Optical Imaging Combined with Radiomic Analysis for Fibrotic Cardiac Tissue Investigation

**DOI:** 10.1021/acs.analchem.5c01510

**Published:** 2025-07-05

**Authors:** Arno Krause, Gabriel Giardina, Laszlo Papp, David Haberl, Clemens P. Spielvogel, Richard D. Walton, James Marchant, Nestor Pallares-Lupon, Kanchan Kulkarni, Xu Li, David L. Vasquez, Jürgen Popp, Iwan W. Schie, Wolfgang Drexler, Marco Andreana, Angelika Unterhuber

**Affiliations:** ‡ Center for Medical Physics and Biomedical Engineering, 27271 Medical University of Vienna, Waehringer Guertel 18-20, 1090 Vienna, Austria; § Department of Biomedical Imaging and Image-guided Therapy (Division of Nuclear Medicine), Medical University of Vienna, Waehringer Guertel 18-20, 1090 Vienna, Austria; ∥ IHU Liryc, Univ. Bordeaux, INSERM U 1045, CRCTB, F-33000 Bordeaux, France; ⊥ Department of Spectroscopy and Imaging, 40096 Leibniz Institute of Photonic Technology (Leibniz-IPHT), Albert-Einstein-Str. 9, 07745 Jena, Germany; # Institute of Physical Chemistry (IPC), Abbe Center of Photonics (ACP), Friedrich-Schiller-University Jena, Helmholtzweg 4, 07743 Jena, Germany; ○ Department of Medical Engineering and Biotechnology, University of Applied Sciences - Jena, Carl-Zeiss-Promenade 2, 07745 Jena, Germany

## Abstract

Understanding the process of fibrotic scarring of the myocardium is critical for the diagnosis and risk stratification of life-threatening cardiac dysfunction. Complex changes in structure, composition, and conductivity occurring at different stages of fibrogenesis diversify the biomedical characteristics of the myocardium. We present a multimodal optical imaging approach including cardiac optical mapping (COM), optical coherence tomography (OCT), multiphoton microscopy (MPM), and line scan Raman microspectroscopy (LSRM) for multiparametric assessment of the myocardium with radiomic analysis to link electrophysiologic, morphologic, functional, and molecular changes in ischemic cardiac tissue and validate our results with histology. COM is used to map the electrical behavior across myocardial tissue. Second harmonic generation and two-photon excitation fluorescence imaging as MPM techniques provide additional unique contrast of collagen, the extracellular matrix, and cardiac cells, such as cardiomyocytes playing a critical role in cardiac fibrosis. Our machine learning model based on radiomic features extracted from MPM data addresses the need for automated fast high-throughput classification between healthy and pathologic cardiac tissues and achieved an accuracy of 0.99. In addition, LSRM assesses the molecular contrast and is used to evaluate the development stage of fibrotic scarring and multiclass classification by utilizing partial least-squares discriminant analysis, achieving sensitivity and specificity values of 0.94. OCT is used for fast navigation through the sample, for intermodal referencing, and easy coregistration between the complementary imaging techniques operating at different fields of view and resolutions ranging from cm^2^ down to μm^2^.

## Graphical abstract

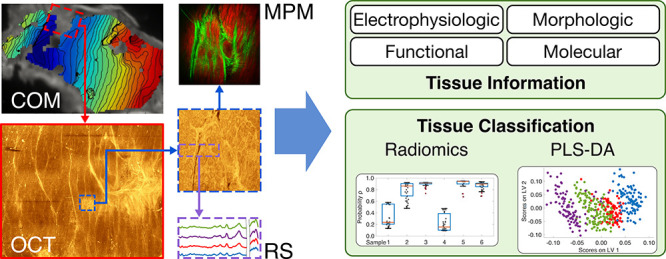

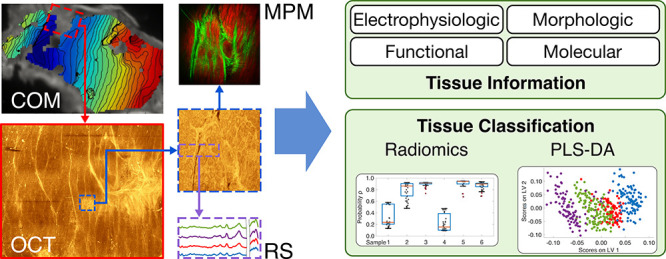

Cardiovascular diseases (CVDs) are a leading cause of mortality worldwide, accounting for approximately one-third of all deaths.^1^ Ischemic heart disease and endomyocardial fibrosis have been identified as primary contributors to end-stage heart failure.^2^ In most cases, fibrotic scarring in cardiac muscle is a result of the repair process after myocardial infarction (MI) accompanied by permanent loss of cardiomyocytes and the formation of a fibrotic scar, predominantly made up of collagen types I and III, leading to increased myocardial stiffness and electrical impairment.^3^ The risk of lethal arrhythmias and heart failure is greatly increased upon clinical signs of cardiac structural remodeling. Abnormal extracellular matrix (ECM) composition and distribution are mainly attributed to collagen secretion in cases of heart failure.^4^ This process correlates with the activation of cardiac fibroblasts and their differentiation into myofibroblasts.^5^ Although there is already an advanced understanding of the composition of the cardiac muscle in health and disease, fundamental knowledge about the mechanisms underlying cardiac fibrosis development and the interaction between extracellular and cellular components with the surviving myocardium and electrical function is still missing. The high mortality associated with CVD creates an urgent need to enhance our understanding of the biology and underlying mechanisms of CVD to develop innovative diagnostic tools and effective treatment.^3^ Current clinical markers, such as ejection fraction and scar surface area, are poor indicators of risk for sudden cardiac death. Scar formation, distribution, and interaction with surrounding myocardium are likely more relevant factors to determine arrhythmia vulnerability. Current clinical imaging tools and strategies integrating microscale cardiac remodeling are lacking.^6^

Cardiac optical mapping (COM) has become an important tool in preclinical research to provide high spatiotemporal resolution images of the electrophysiology of the heart utilizing fluorescent dyes.^7^ However, the need to develop fluorescent dyes with optimized spectral properties, considering experimental time scales, ensuring biological compatibility for in vivo imaging, as well as visualization and interpretation of the signal, can be challenging since the tissue might be altered by the dyes.

In recent years, the development of various label-free and noninvasive optical imaging techniques, such as optical coherence tomography (OCT), multiphoton microscopy (MPM), and Raman spectroscopy (RS) have emerged in the biomedical and diagnostic fields and go far beyond simple visualization of the sample.^8–10^ OCT as an interferometric technique offers fast volumetric ultrahigh resolution morphologic information based on changes in the refractive index. The potential of OCT as virtual histology of the human heart to provide high-resolution images of cardiac tissue and to discriminate between tissue types such as healthy muscle, adipose, and fibrotic tissues has been shown.^11^ MPM provides confocal high-resolution three-dimensional (3D) information about tissue morphology and molecular composition with reduced photobleaching and phototoxicity as well as intrinsic 3D sectioning and enhanced penetration depth compared to traditional confocal microscopy. Myocardium consists of highly birefringent, carefully aligned bundles of cardiomyocytes surrounded by the complex protein networks forming the ECM.^12^ Changes in the collagen content, as primary protein of the ECM, can be investigated with second harmonic generation (SHG) microscopy.^13^ In addition, two-photon excitation fluorescence (TPEF) microscopy provides a specific contrast of cardiomyocytes allowing imaging of the cell morphology. However, until now, advanced automated quantitative methods to analyze MPM images are lacking, and radiomic analysis approaches for feature extraction and classification are in their infancy.^14^ Radiomic analysis has been established as a high-throughput method to extract large amounts of image features from radiographic images in an automated and quantitative manner to identify potential biomarkers that are descriptive for a given disease.^15^ Its implementation is still limited to some proof-of-concept clinical computed tomography and magnetic resonance studies to demonstrate the potential, diagnostic performance, and identification of relevant biomarkers for a given pathologic state.^16,17^ RS provides highly specific molecular information about tissue composition and has been used to classify the different stages of fibrotic scarring.^18^ By applying regression analysis, such as partial least-squares discriminant analysis (PLS-DA), prediction models can be built that relate variations of spectral Raman data to the supervised sample groups by transforming the data set to a set of latent variables (LVs).^19^ Regression analysis can be used to distinguish between different sample groups.

Current research efforts have shown that a single modality does not provide enough information to understand a disease comprehensively. A carefully selected combination of noninvasive optical modalities, enabling access to a diverse set of parameters, is necessary. Multimodal optical imaging, particularly the synergistic combination of OCT, MPM, and RS, has been introduced to integrate complementary contrast mechanisms and overcome the limitations of individual modalities.^20,21^

In this work, we combined noninvasive multimodal optical imaging techniques with radiomic analysis to get deeper pathophysiologic insights into the event of fibrotic scarring after MI, which might help to better understand the arrhythmogenic mechanisms of underlying MI, establish new diagnostic tools, and improve current treatment strategies. We used COM for cardiac tissue examination on a macroscopic level and combine the information with ultrahigh-resolution spectral domain OCT, MPM, and line scan Raman microspectroscopy (LSRM) as advanced optical imaging modalities on a microscopic level. Seamless intra- and intermodal image coregistration provided complementary electrophysiologic, morphologic, functional, and molecular 3D tissue information on intact tissue down to the cellular level and was directly correlated with Masson’s trichrome staining (MT). Quantitative radiomic analysis of MPM images and multivariate analysis of Raman data with PLS-DA demonstrated the capability to classify between healthy tissue and different pathologic states by extraction of relevant features as biomarkers, which are descriptive for cardiac fibrosis.

## Materials and Methods

### Sample Preparation and Masson’s Trichrome Staining

The experimental study included hearts obtained from an ovine model with chronic MI. Left ventricles were dissected from harvested hearts, and 400 μm thick slices covering an area of about 20 × 25 mm from different locations from the left ventricles were used for all experiments. For further information about sample tissue preparation and sample mounting see Supporting Information I. Consecutive tissue slices were used for comparison with COM and multimodal high resolution optical imaging. After imaging, MT was performed according to a standard protocol for identification of collagen fibers (blue), nuclei (black), and muscles, cytoplasm, and keratin (red). The background appeared in red. Slides were scanned and annotated by a trained pathologist into four tissue types: healthy, necrotic, granulated, and fibrotic. The cases of necrotic, granulated, and fibrotic tissue are summarized as pathologic tissue.

### Multimodal Imaging

Our multimodal imaging pipeline was designed for multiparametric cardiac tissue investigation of sample pairs after MI, from the macroscopic level down to the microscopic level, as shown in Figure 1. In the first step, COM was performed on the whole slides to generate epi-fluorescent low-resolution large field of view (FOV) images from voltage changes to extract information about cardiac electrophysiology. COM was then correlated via the low-magnification OCT mode to the other part of the sample pair on cm^2^ FOVs. Switching to a high-magnification OCT mode on our multimodal noninvasive optical imaging platform featuring OCT, MPM, and LSRM already described elsewhere allowed for intermodal referencing on μm^2^ FOVs.^14^ In this step of our pipeline, the customized upright laser scanning microscope provided a common sample path for all modalities to facilitate image coregistration. Multiscale OCT was used for fast tissue scanning and intermodal referencing. In detail, fast 3D widefield navigation through the tissue was performed in low-magnification OCT mode with easy intermodal correlation to high-magnification OCT mode.

**Figure 1:**
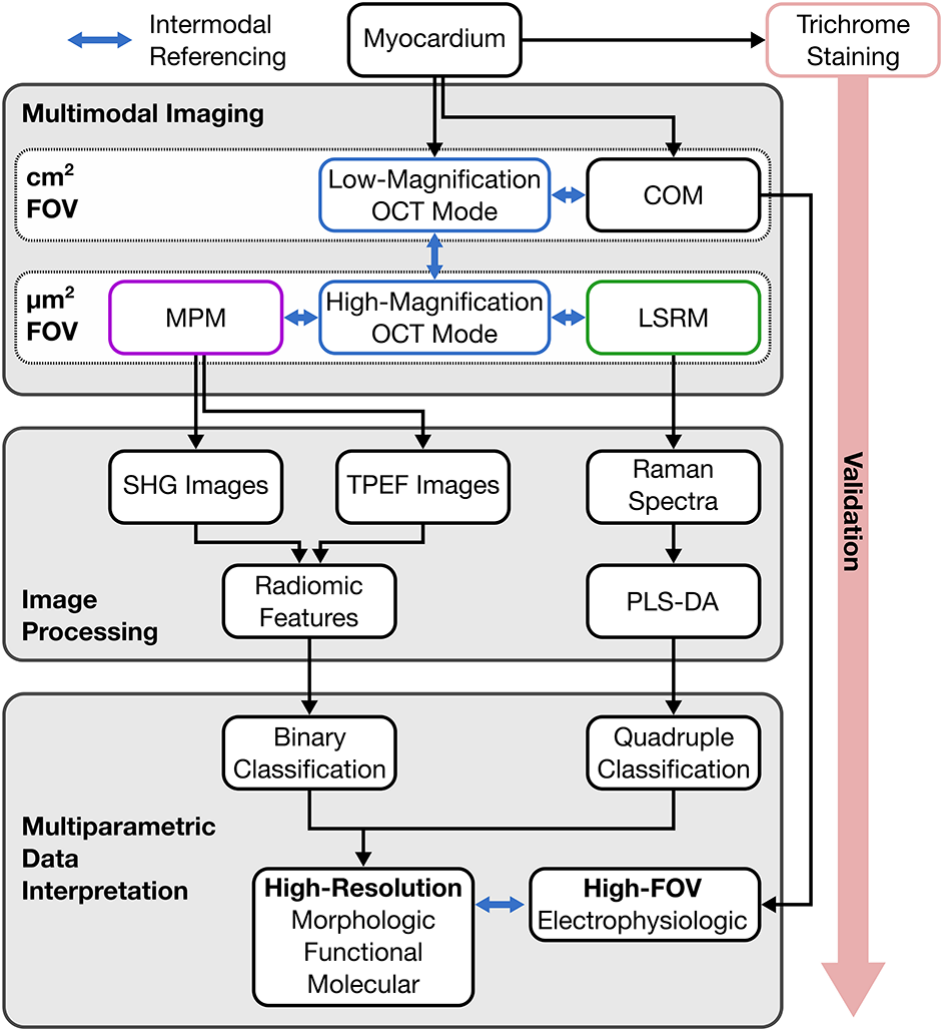
Workflow for myocardial analysis, integrating COM, OCT, MPM, and LSRM. Image processing includes radiomics of label-free SHG and TPEF images and PLS-DA of Raman spectra, for multiparametric data interpretation at different magnifications.

The same objective was used for MPM imaging acquired in two different channels (SHG, TPEF) simultaneously as well as LSRM to ensure easy coregistration between the OCT, MPM, and LSRM and correlation with COM and histology to directly link and combine complementary electrophysiologic information to morphologic, functional, and molecular information on FOVs from hundreds of μm^2^ to cm^2^. In our study, we investigated nine regions of interest (ROIs) around the areas of the MI-induced scar. The most important parameters of each modality can be found in the Supporting Information II (see Table S1).

### Image Processing

For radiomic analysis, images of each MPM modality were preprocessed in terms of binning, masking, and normalization. SHG and TPEF image data within the mask was subject to radiomic analysis and used for training, validation, and testing of the radiomics machine learning (ML). A binary classification between healthy and pathologic tissue was chosen. Radiomic features, standardized according to the Imaging Biomarker Standardization Initiative (IBSI), were extracted from each multimodal image using the MUW radiomics engine (version 2.0).^15^ In the case of Raman spectra, dimensionality reduction was performed with PLS-DA and scores and loadings of first and second latent variable (LV) were calculated. The same data set was used for the direct multiclass classification using an open-source PLS-DA MATLAB toolbox.^22^ The PLS-DA toolbox parameters and further information about the radiomics ML can be found in the Supporting Information II and III, respectively.

### Multiparametric Data Interpretation

Quantitative radiomic analysis on SHG and TPEF data and multivariate analysis with PLS-DA on Raman data were performed for binary and quadruple tissue classification with high resolution, respectively, and linked to large FOV electrophysiologic information. The proposed combination of COM, OCT, MPM, and LSRM enables the acquisition of highly resolved morphologic, functional, and molecular information linked via OCT to high-FOV electrophysiologic information. The multiparametric information from the multimodal imaging pipeline was validated with MT as a gold standard technique.

## Results and Discussion

The results are organized in subsections of the multiscale multimodal analysis of the myocardium and the multiparametric biomarkers obtained from the ultrahigh resolution noninvasive multimodal coregistered images.

### Multimodal Imaging Pipeline

During COM, tissue undergoes prolonged superfusion with physiological saline solutions, edema, and chemical electromechanical uncoupling, which often leads to morphologic alterations, such as shrinkage and curling of the tissue slice borders. These changes are obvious when comparing a cardiac tissue sample following optical mapping versus the adjacent sample that was rapidly fixed for direct OCT, MPM, and LSRM, as shown in Figures 2d and 2a, respectively. The color variation between samples is mainly attributed to tissue staining by fluorescent voltage-sensitive dyes used to measure electrical activity in COM. The presence of exogenous fluorescent markers severely alters the optical properties of the sample; therefore, we always used sample pairs, where one sample was investigated with COM and the neighboring sample with label-free noninvasive high-resolution techniques such as OCT, MPM, and LSRM which do not cause any tissue alterations. In that way, it could be ensured that the alterations due to COM did not influence the multiparametric data interpretation. Figures 2e and 2j show the COM results with the activation time (AT) map at 3 and 1.5 Hz pacing, respectively. Stressing the tissue sample through pacing at 3 Hz imposes regions of slow conduction, notably in regions with reduced electrical coupling by fibrosis. Figure 2j shows an unexcitable region being circumnavigated by an electrical wavefront, a prerequisite of arrhythmic re-entrant circuits, indicated by the yellow arrow. Another area with abnormal electrical conductivity can be clearly identified in both AT maps by the absence of fluorescent emission and is indicated with red dashed rectangles in Figures 2e and 2j. This area was further analyzed with noninvasive label-free ultrahigh-resolution multimodal imaging and histologic examination with MT as part of our proposed pipeline as shown in Figure 2b (low-magnification OCT), Figures 2f, 2g, 2k, and 2l (high-magnification OCT), Figures 2h and 2m (MPM), and Figures 2c, 2i, and 2n (MT). OCT provided fast 2D and 3D information about tissue morphology but was less specific than MPM or LSRM providing functional and molecular information. Once a ROI was identified by OCT at low magnification, a high-magnification mode was applied and switched to MPM. However, since muscles and collagen tissue could also be discriminated in the OCT images due to changes in the refractive indices, OCT could be used to prescreen the ischemic part and to identify fibrotic areas.^11^ Textural features, such as microvasculature and collagen fibers, are indicated in Figure 2 with white and red arrows, respectively, and can be traced via OCT to MPM and LSRM and demonstrate the potential of OCT for fast widefield prescreening and multiscale intermodal referencing. Bright fibrillar structures are linked to high densities of collagen present in cardiac fibrosis as shown in the OCT en face representations in Figures 2b, 2g, and 2l and validated with MT in Figures 2c, 2i, and 2n. A big collagen cluster is visible in the low-magnification OCT image indicated with a black arrow in Figure 2b, which correlates to the MT in Figure 2c where high amounts of collagen indicative for fibrotic tissue are visible. Cardiomyocytes are mostly replaced by collagen. Residual inflammatory cells (black dots) indicate the late-state fibrotic scarring. The gradual color change from blue to blue/red and further to pure red of the surrounding tissue showed the transition from pathologic necrotic to healthy muscle tissue.^23^ Due to fixation and cutting required for MT, imperfections are found when overlaying images between modalities. The MT image shows some voids and ruptures where information is missing due to the tissue sectioning process together with shrinkage from the preservation process. Despite this limitation, overlays were adequate between low-magnification OCT and MT. The overall morphology within the blue solid box in Figure 2b appears more homogeneous and less bright than the morphology within the dashed blue box, where bright spots can be linked to the high collagen content in pathologic conditions. High-magnification OCT en face planes (Figures 2g and 2l) and B-scans (Figures 2f and 2k) were acquired at the location of the boxes and show that textural features can be better resolved with higher resolution especially at the transition between fibrotic tissue induced by the scarring process after MI and the surrounding tissue. The morphology is directly validated with MT via low-magnification OCT. The en face high-magnification OCT planes in Figures 2g and 2l show the morphology from muscle tissue and a fibrotic cluster at depths of about 50 μm into the tissue, as indicated in the corresponding B-scans with blue lines. The coregistered 2D MPM planes in Figures 2h and 2m confirmed the collagen deposition at these sites and revealed additional, specific morphologic contrast on the same FOVs. The bright spots in the OCT images correspond to a high collagen content in MPM images. Figures 2i and 2n show the corresponding MT images and confirm the tissue in the solid blue box as muscle and in the blue dashed box as fibrotic tissue with a large number of cardiomyocytes and a collagen network showing increased ramification next to altered muscle tissue.

**Figure 2:**
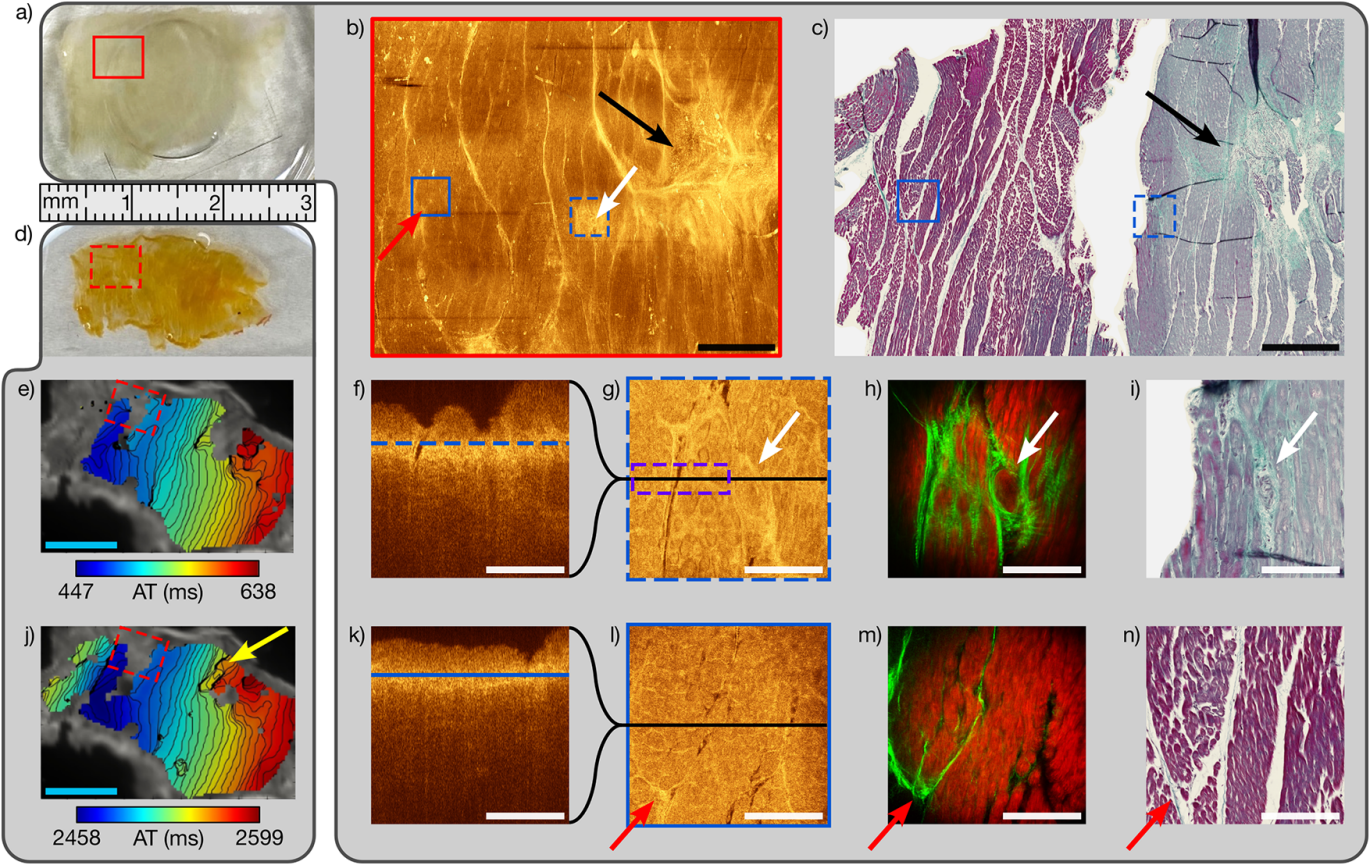
Multiscale multimodal analysis of a tissue pair (a, d) from a scar border region from the LV myocardium. (a) Cardiac tissue after noninvasive multimodal optical imaging performed on the red rectangle. (b) Low-magnification mode maximum intensity projection of the OCT image. (c) MT image of (b). Black arrows point to fibrotic tissue visible in the OCT and MT. Dashed and solid blue rectangles show the position of high-magnification OCT scans (g) and (l), respectively. (f) and (k) High-magnification OCT B-scans with blue lines showing the depth position of the OCT images in (g) and (l), respectively. Black lines in (g) and (l) show the lateral position of the OCT B-Scans (f) and (k), respectively. The purple rectangle in (g) shows the area scanned by LSRM. (h) and (m) MPM images with SHG channel (green) and TPEF channel (red) coregistered with the OCT images (g) and (l) and validated with MT in (i) and (n). (e) and (j) AT maps at pacing frequencies of 3 and 1.5 Hz, respectively. Isochrones are spaced 5 ms. The yellow arrow points to a possible re-entrant circuit. White arrows indicate a microvascular structure, and red arrows indicate a triangular-shaped collagen formation. White scale bars 200 μm, black 1 mm, and blue 5 mm.

### Multiphoton Microscopy and Radiomic Analysis

In healthy myocardium, cardiomyocytes are well-aligned and densely arranged in laminae with only sparse evidence of collagen fibers between laminae, as shown in Figures 2m and 3a (healthy).^24^ The MPM signal offers simultaneously epi-detected SHG contrast from one channel (green) mainly arising from collagen fibers and TPEF contrast from the second channel (red) arising from autofluorescence of cardiomyocytes in addition to the less specific morphologic information from the OCT. Under MPM, the dense and tight arrangement of healthy myocardium results in dominant autofluorescence contributions to the image (red TPEF channel). Only a few thin collagen fibers are visible between the myocardial fiber bundles in the green SHG channel. These thin fibers can also be seen in the MT appearing as very faint blue lines as shown in Figures 2n and 3a (healthy). The SHG-sensitive collagen forms a major component of the cardiac ECM, functioning to limit the sheer stress between muscle laminae during contraction of the heart. Figure 3a (pathologic/necrotic) shows typical multimodal MPM planes of the necrotic tissue. Although signal from the TPEF channel indicates laminae of surviving cardiomyocytes, there is an evidently elevated SHG signal throughout the myocardium. The SHG channel shows a higher amount of mainly long and curled collagen fibers. The overall TPEF signal is reduced compared to the healthy state.

**Figure 3:**
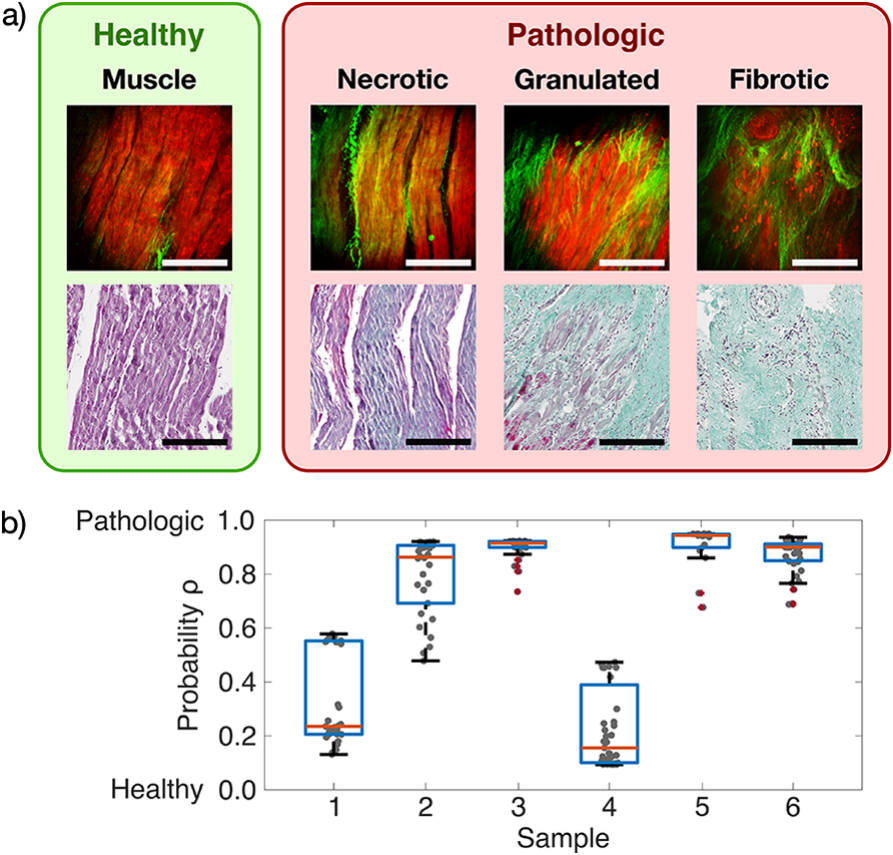
Coregistered optical imaging of infarcted cardiac samples with radiomic analysis based on MPM contrast. (a) Representative MPM images used for training and testing of the ML model (upper row) for binary classification with the corresponding MT images (lower row). The SHG channel is colored in green, and the TPEF channel is colored in red. Scale bars 200 μm. (b) Box plot of ML training shows probability values ρ of each MPM image.

MPM can therefore resolve necrotic tissue that shows retained muscle fiber organization, but with considerable invasion of collagen into the interstitium. Granulation shows a later stage of the scarring process where the fibrillar collagen replaces the myocardium, as indicated in Figure 3a (pathologic/granulated). Cardiomyocytes are still visible in the TPEF channel but increased discord of cellular arrangement, and the space between laminae is rich with long and curled collagen fibers appearing in the SHG channel. Collagen fibrils have greater thickness and are intertwined in a complex array, instead of running parallel to muscle laminae. The granulated SHG signal is higher compared to the healthy and necrotic states. The corresponding MT shows residual necrotic muscle tissue appearing in blue/red and replacement fibrosis appearing in blue.^25^ Inflammatory cells visible by the dark spots of the cell nuclei indicate an ongoing scar formation process. Fibrotic tissue in Figure 3a (pathologic/fibrotic) shows a much lower TPEF intensity compared to that of the healthy state but also compared to that of the granulated tissue. Cardiomyocytes are almost entirely replaced by fibrosis, and the few surviving myocytes are separated by collagen deposits, exacerbating cellular uncoupling, as confirmed by MT. The SHG contribution in the fibrotic tissue is much higher, and thick and long intertwined collagen fibers that form a mesh are visible. Granulated and fibrotic tissues can be characterized by a dense randomly oriented mesh of collagen and nonuniform muscle fibers visible as loosely arranged cardiomyocyte fibers and long intertwined collagen fibers.^26^ This collagen mesh formation in the scarring process after MI helps to increase the mechanical stability and is a hallmark of myocardial scarring.

We used an ML model based on radiomic feature extraction to obtain an objective, reproducible, and quantitative analysis of the obtained imaging features and classify MPM images into healthy or pathologic tissue. In total, 562 SHG and TPEF images were used. 184 planes acquired from 3 ROIs built the training and validation data set. The binary classified training set was set by 56 healthy and 128 pathologic planes representing muscle tissue and necrotic, granulated, and fibrotic tissues in the histopathologic classification, respectively, as shown in Figure 3a. 152 IBSI-conform radiomic features were extracted for SHG and TPEF, respectively, resulting in 304 radiomic features. To reduce the probability of overfitting, the features were ranked with minimum redundancy maximum relevance and the nine highest-ranked features were used for the training and validation set of each MC fold satisfying *d* ≈ √*n* with optimal feature size *d* and sample size *n*.^14^ To ensure permanent data balancing in each MC fold, 20% in the validation data set was reflected on the minority group size (healthy). The same number of samples was taken from the majority group (pathologic), while all remaining samples were used for the training data set. Data balancing in the training data set was achieved with a synthetic minority oversampling technique by synthetic oversampling of the healthy group. Each MC fold was unique, preventing repeated subset configurations. All performance values were calculated across each MC fold, resulting in accuracy values of at least 0.99 and sensitivity and specificity values of at least 0.98. For testing, the predictions (votes) of each ML classifier were aggregated, and the class with the majority of votes was selected as the final output. 378 SHG and TPEF images from six ROIs were used for testing. For prediction, whether a sample belongs to a pathologic or healthy state, the mean ρ was calculated for each multimodal image by averaging the prediction values from all ML classifiers and illustrated as a box plot with each cardiac sample as shown in Figure 3b. Samples 1 and 4 in Figure 3b show representative healthy muscle tissue. Samples 2 and 3 are both classified as necrotic tissue, but while sample 3 shows high probability values toward pathologic tissue for all sample positions, the probability values for sample 2 range from medium to high depending on the depth. From the five highest-ranked features from the radiomic analysis shown in Table 1, the first three are attributed to differences in SHG contrast. While the features short runs emphasis (SRE), high dependence high gray level emphasis (HDHGE), and texture strength and local intensity peak from the feature groups gray level run length matrix (GLRLM), neighboring gray level dependence matrix (NGLDM), neighborhood gray-tone difference matrix (NGTDM), and intensity feature group are attributed to SHG, the intensity range feature from the intensity-based feature group is attributed to TPEF.^27^ A measure of the well-organized, linear collagen networks representing healthy cardiac tissue can be provided by low SRE values from GLRLM.^28^ High SRE values are displayed by pathologic tissue due to fragmented ECM structures. The spatial rate of the textural change is measured with NGTDM features, observing the coarseness and structures emphasized from the background. In the case of the ECM, a high texture strength value corresponds to a larger structure emphasized from the background in the image, such as a high amount of bundled collagen. The fourth most important feature, as intensity range, is the only significant feature obtained from the TPEF images and emphasizes the importance of collagen as biomarker for cardiac fibrosis.

**Table 1:** Five Highest Ranked Features Resulting from the Radiomics-Based Machine Learning

Modality	Feature Name	Group	Mean Ranking (%)	Description
SHG	Short runs emphasis	GLRLM	7.24 ± 2.31	Length of consecutive pixel sequence
SHG	High dependence high gray level emphasis	NGLDM	6.14 ± 0.62	High similarity of high gray level neighboring pixels
SHG	Texture strength	NGTDM	5.47 ± 0.17	Measure of large coarse differences in gray level intensities
TPEF	Intensity range	Intensity	4.71 ± 1.79	Measure of intensity range
SHG	Local intensity peak	Intensity	4.67 ± 1.96	Mean intensity around maximum intensity level

In our study, the masks used for radiomics training are created by an OR condition of individual masks from SHG and TPEF images, respectively. Since the TPEF signal extends laterally far beyond the SHG signal, background noise is also encountered in the SHG channel for ML. To evaluate the reliability of an OR-combined automated masking, a separate training session was performed using the present TPEF and mask images, along with manually threshed SHG images. For more details about the image masking, see Supporting Information III.1. The SHG images with a more emphasized fibrillar structure were used for the training. The performance values with accuracies of 0.99, sensitivities of 1.00, and specificities of at least 0.96 were achieved through all classifiers, which is similar to the performances when fully automated masks and unchanged SHG images are used. Further prediction performances are listed in Tables S2 and S3. Comparing the performance values from automated and manual masked SHG data in Tables S2 and S3, we find that the overall accuracy through all classifiers is similar. The overall performance in positive and negative predictions is outstanding. The prediction value of an MPM image was determined by averaging the output values from all classifiers. This did not require hyperparameter optimization and, if applicable, ML model selection by an additional validation set. However, considering the employed ML classifiers, the benefit of manual masked SHG signals over automated masks cannot be confirmed. The results underline the importance of collagen as a biomarker and are in good agreement with the literature where SHG was used for classification of examined cardiac tissue into noninfarcted, pharmacologic treated, and untreated based on density and individual fibril morphology of collagen.^29^ In the study of Lagarto et al., autofluorescence intensity and lifetime parameters of flavin adenine dinucleotide and reduced nicotinamide adenine dinucleotide were identified as markers for functional changes caused by oxygen depletion, which is an essential trigger for fibrosis.^30^ In our approach, we used fixed tissue samples and therefore did not concentrate on functional changes but were interested in changes of cardiomyocyte organization as a potential biomarker. In that way, we did not need to overcomplicate the MPM system and measurements by considering the lifetime parameters. However, radiomic analysis showed that not only collagen could be identified as an important biomarker but also intensity changes in the TPEF signal related to contrast of cardiomyocytes. Therefore, the combined TPEF and SHG contrast could be used to efficiently train ML models to perform fast automated binary classification of cardiac samples as either healthy or pathologic, even in cases of varying stages of fibrosis.

### Line Scan Raman Microspectroscopy Results

For multiclass classification, we performed LSRM. Due to long acquisition times required for RM signal collection, LSRM was only performed on a limited FOV for all samples as indicated with a magenta box in Figure 2g. RS reveals subtle molecular changes by observing different vibrational modes. Mean spectra with corresponding standard deviation for muscle, necrotic, granulated, and fibrotic tissues are shown in Figure 4a. In the fingerprint region characteristic peaks could be assigned to amide III at 1260 cm^–1^, CH_2_ and CH_3_ wagging modes of collagen at 1338 cm^–1^, CH_2_ and CH_3_ deformation modes of collagen at 1446 cm^–1^, and amide I with collagen assignment at 1650 cm^–1^.^31^ The rearrangement of collagen fiber bundles due to fibrotic scarring causes variations in the Raman bands of amide I 1625–1700 cm^–1^ and amide III 1200–1300 cm^–1^.^32^ Especially the amide I band can be linked to collagen fiber packing and changes in the connectivity and shows distinct differences compared to intensity variations at 1650 cm^–1^, confirming our MPM radiomics results that have identified collagen as a prominent biomarker for cardiac fibrosis and also in perfect agreement with previous studies reporting that main changes in the Raman spectra are attributed to collagen.^18,33^ Changes in the asymmetric CH_2_ vibration modes at 2880 cm^–1^ and symmetric CH_3_ stretching vibrations at 2920 cm^–1^ are attributed to lipid and protein variations of myelinated and unmyelinated nerve fibers.^34^

**Figure 4:**
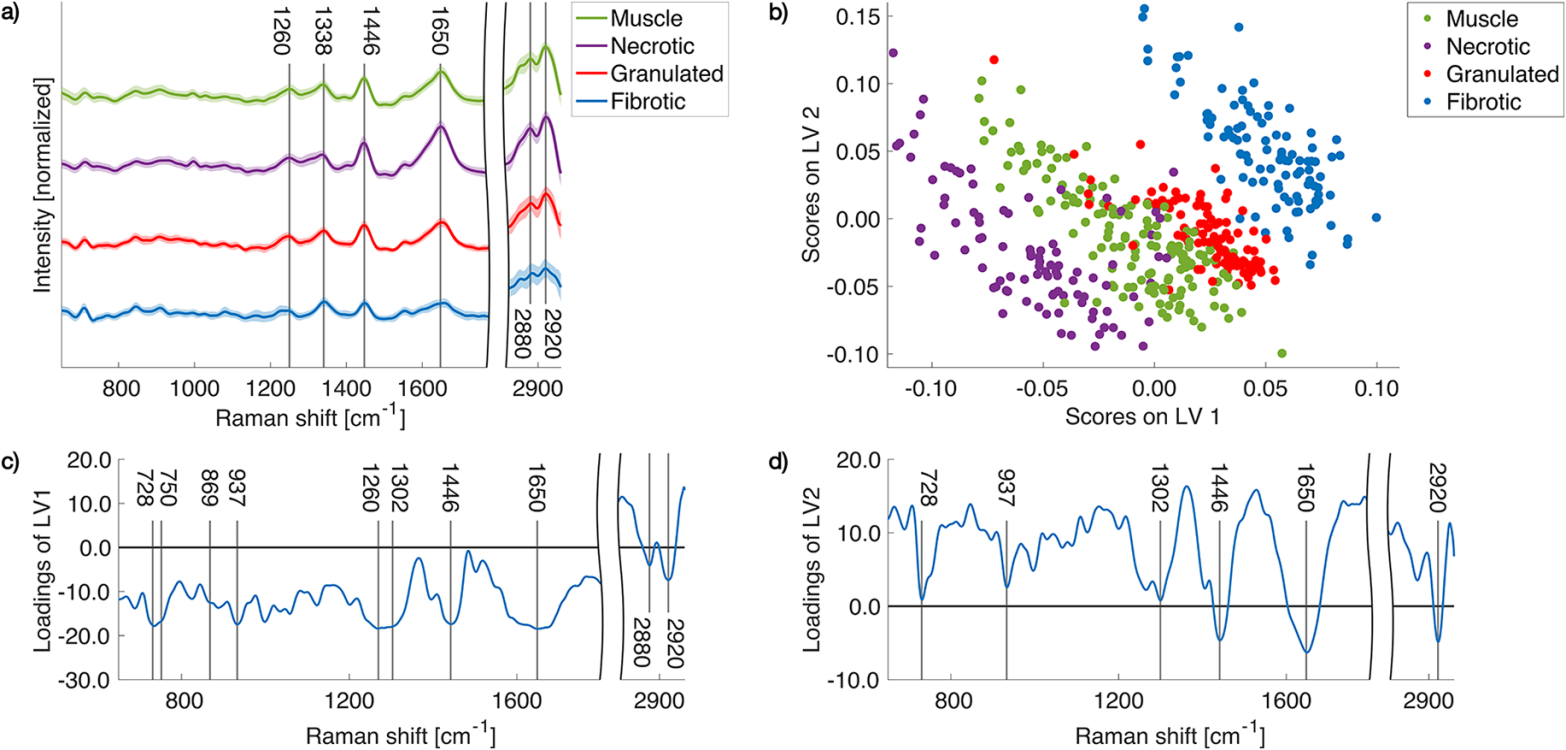
Multiclass Raman spectroscopy results: (a) Mean Raman spectra from muscle, necrotic, granulated, and fibrotic tissues. (b) PLS-DA score plot of LV1 vs LV2 showing clustering of necrotic, muscle, granulated, and fibrotic samples. (c) Loadings of LV1 and d) LV2.

To perform multiclass analysis and reduce the dimensionality of the processed spectra, the LVs are calculated using PLS-DA. The PLS-DA score plot of the first (LV1) versus the second latent variable (LV2) in Figure 4b shows the clustering of the spectral components into necrotic, muscle, granulated, and fibrotic tissues, respectively. The loadings of the first and second LVs derived from the PLS-DA, as shown in Figures 4c and 4d, reveal distinct differences in the spectral components of the different tissue types and are used to discriminate the tissue types and provide important information about the molecular changes. The most eminent features of the LV1 are the peaks at 1446 cm^–1^ and 1650 cm^–1^ assigned to CH_2_ and CH_3_ deformation and amide I, respectively, all of which are assigned to collagen type I. The C–C stretching of proline at 728 cm^–1^, amide III at 1260 cm^–1^, and the peaks of the twisting, wagging, and bending modes of CH_2_ at 1302 cm^–1^, all with collagen assignment, underline the importance of collagen during fibrosis. C–C stretching and vibrations of the choline group of collagen and lipids is observed at 869 cm^–1^ and C–C vibration of collagen backbone is shown at 937 cm^–1^.^35^ The peak at 750 cm^–1^ corresponds to c-type and b-type cytochromes.^36^ The peaks of the different tissue types in the C–H stretching region show the same trend as the peaks in the fingerprint region with an intensity decrease for the peaks at 2880 cm^–1^ and 2920 cm^–1^ during the transition from muscular to fibrotic tissue. Our trained algorithm achieved outstanding performance with group specificity values for muscle, necrotic, granulated, and fibrotic tissues of 0.95, 0.99, 0.98, and 0.99, respectively, and sensitivity values of 0.94, 0.98, 0.87, and 0.97, respectively. The overall sensitivity and specificity values are 0.94, respectively. The major role of collagen during fibrosis, in which myofibroblasts are replaced in a first stage by collagen type III and later by collagen type I, is clearly shown in our study by comparing mean spectra and the subsequent analysis with PLS-DA.^37^ Within a time span of days to weeks after the MI, collagenous scar tissue gradually replaces dead cardiomyocytes.^38^ The entire transformation process into a hypocellular compact scar, with dilated thin-walled vessels, takes up to a couple of months. Our multiscale multimodal imaging pipeline may enable a better understanding of the molecular contributions and influence of the various collagen types in cardiac pathogenesis. Notably, collagen type I is augmented for MI-related fibrotic scarring, whereas ischemic cardiomyopathy causes increased deposition of collagen type III.^6^ Since RS is a powerful tool to identify structural changes of collagen, ratios of the collagen-related vibrational Raman bands may leverage standardized analytical approaches to investigate the fibrotic process.^39^

## Conclusions

Our noninvasive and label-free multimodal optical imaging platform in combination with COM and validation with MT provides comprehensive information on unaltered myocardial tissue during fibrotic scarring. COM disclosed abnormalities in electrical conductivity of the myocardium, pointing to fibrotic scars and re-entry circuits. OCT enabled 3D real-time observation of tissue structure in low- and high-magnification mode facilitating the intra- and intermodal correlation of fibrotic areas. Multimodal SHG and TPEF images coregistered with the OCT offered additional detailed 3D insights into the collagen structure and organization of the ECM and cardiomyocytes as crucial parameters to study the structural changes associated with fibrosis, where collagen deposition and reorganization are key features. In combination with radiomic analysis, these nonlinear techniques allow for the precise quantification of collagen density, orientation, and distribution, providing detailed information on the extent of fibrosis and automated classification into healthy and pathologic tissues. Multiple ML models were trained and fully implemented in our multimodal optical imaging workflow, underscoring the crucial role of collagen as a biomarker. Moreover, LSRM provided direct fast access to molecular composition analysis on a reduced FOV revealing excessive accumulation of collagen and variations of other ECM components such as proteins, lipids, and nucleic acids, which are indicative of fibrosis and enable quantification and mapping of fibrotic tissue with automated multiclass analysis and trained PLS-DA. However, MPM allowed for faster and high-throughput image analysis compared to LSRM. OCT outperforms both techniques in terms of acquisition speed but offers less specific contrast. LSRM requires the longest acquisition times, but its ability to identify multiple pathophysiologic classes allows for precise tissue classification within the MPM FOV.

We have demonstrated the potential for a multimodal and multiscale workflow combining advanced optical imaging techniques with radiomic-based ML and regression analysis to provide a comprehensive assessment of cardiac fibrosis, helping to elucidate disease-specific patterns of fibrosis for a better understanding of the scarring processes after MI and support the development of diagnostic tools and therapeutic strategies in the field of ischemic heart diseases.

## Supplementary Material



## Notes

Sample preparation and handling, system designs and image processing, applied ML models and classifier (PDF)

## †

Sanford Burnham Prebys Medical Discovery Institute, La Jolla, California, 92037, United States
