# Supplementary material for: Multimodal Optical Imaging Combined with Radiomic Analysis for Fibrotic Cardiac Tissue Investigation
Source: Anal Chem. 2025 Jul 5;97(27):14397–405. doi: 10.1021/acs.analchem.5c01510 (PMC12268825; doi:10.1021/acs.analchem.5c01510)
Supplement: Supplementary file 1 [file ac5c01510_si_001.pdf]

Supplemental Materials

Multimodal optical imaging combined with radiomic analysis for fibrotic cardiac tissue investigation

Arno Krause[1], Gabriel Giardina[1], Laszlo Papp[1], David Haberl[2], Clemens P. Spielvogel[2], Richard D. Walton[3], James Marchant[3], Nestor Pallares-Lupon[3], Kanchan Kulkarni[3], Xu Li[1], David L. Vasquez[4], Jürgen Popp[4,5], Iwan W. Schie[4,6], Wolfgang Drexler[1], Marco Andreana[1], and Angelika Unterhuber[1]

[1]Center for Medical Physics and Biomedical Engineering, Medical University of Vienna, Waehringer Guertel 18-20, 1090 Vienna, Austria

[2]Department of Biomedical Imaging and Image-guided Therapy (Division of Nuclear Medicine), Medical University of Vienna, Waehringer Guertel 18-20, 1090 Vienna, Austria

[3]IHU Liryc, Univ. Bordeaux, INSERM U 1045, CRCTB, F-33000 Bordeaux, France

[4]Department of Spectroscopy and Imaging, Leibniz Institute of Photonic Technology (Leibniz-IPHT), Albert-Einstein-Str. 9, 07745 Jena, Germany

[5]Institute of Physical Chemistry (IPC), Abbe Center of Photonics (ACP), Friedrich-Schiller-University Jena, Helmholtzweg 4, 07743 Jena, Germany

[6]Department of Medical Engineering and Biotechnology, University of Applied Sciences - Jena, Carl-Zeiss-Promenade 2, 07745 Jena, Germany

Contents

I Cardiac Tissue Preparation **S2**

II Multimodal Imaging and Processing **S3**

III Radiomic Analysis **S5**

III.1 Image Preparation S5

III.2 Machine Learning Models S7

I Cardiac Tissue Preparation

In accordance with the guidelines of Directive 2010/63/EU of the European Parliament on the protection of animals used for scientific purposes and approved by the Direction générale de la re-cherche et de l'innovation, Cellule Animaux utilisés à des Fins Scientifiques-AFiS, the experimental study included hearts obtained from an ovine infarction model with sheep weighting 40 - 50 kg. The ovine infarction was induced as proposed by *Dib et al.*[1] The animals underwent surgery under general anesthesia to induce anterior-apical MI by deploying embolization coils in the left anterior descending artery, followed by a 6-week recovery period for scar tissue formation. After recovery, the sheep with chronic MI were pretreated with ketamine ($20\,mg/kg$) and acepromazine ($0.02\,mL/kg$) before anesthesia was induced by propofol ($2\,mg/kg$) and kept under isoflurane, 2%, in air/O_2 (50/50%) after intratracheal intubation [2]. Intravenous injection of pentobarbital ($30\,mL/50\,kg$) was used for euthanisation and the cardiac samples were prepared following the protocol from *Pallares-Lupon et al.* [3] Left ventricular myocardial samples were dissected into $2\,\mathrm{cm} \times 2.5\,\mathrm{cm}$ blocks while submerged in cardioplegic solution to preserve structural integrity. A 4% agarose gel, pre-solidified at 4°C, was affixed to the mounting stage of a precision vibrating microtome (7000 smz-2, Campden Instruments Ltd., UK). Tissue blocks were oriented with the endocardial surface facing upward and positioned onto the agarose. The agarose support permitted full exposure of the tissue to superfusion media during slicing. Tissue sections were obtained parallel to the endocardial plane. The vibratome chamber was filled with a cold (4°C), oxygen-saturated (100% O_2) modified Tyrode's solution, supplemented with 10 mM 2,3-butanedione monoxime (BDM) to inhibit excitation-contraction coupling. The solution composition was as follows (in mM): NaCl 140, KCl 6, glucose 10, HEPES 10, $MgCl_2$ 1, $CaCl_2$ 1.8, BDM 10; adjusted to pH 7.4. Slicing parameters were configured to a section thickness of $380\,\mu m$, with an advancement rate of 0.02–0.03 mm/s, a vibration amplitude of 2 mm, and a frequency of 80 Hz. Prior to use, the ceramic blade was calibrated to ensure the vertical (z-axis) vibration remained below $0.5\,\mu m$, minimizing disruption to individual cardiomyocyte layers.
Immediately following sectioning, each slice was transferred to a 20°C oxygenated recovery solution containing the following (in mM): NaCl 140, KCl 4.5, glucose 10, HEPES 10, $MgCl_2$ 1, $CaCl_2$ 1.8, supplemented with 1% Penicillin-Streptomycin, adjusted to pH 7.4. Slices were placed within $100\,\mu m$ nylon mesh cell strainers seated in six-well plates with perforated bottoms to permit continuous oxygenation. A secondary mesh washer was gently placed atop each slice to prevent tissue deformation or curling. The slices were incubated in the recovery solution for at least 20 minutes to allow gradual warming to ambient temperature and to facilitate BDM clearance from the tissue.
After imaging, tissue slices were fixed and embedded in paraffin, sectioned to thicknesses of $5\,\mu$m parallel to the imaging planes, and subsequently evaluated with MT to validate the observed tissue microstructure and composition. For histopathologic classification, MT was performed according to a standard protocol and the slides were scanned using an automated microscope (Axio Scan Z.1, Carl Zeiss AG, Oberkochen, Germany).

II Multimodal Imaging and Processing

The system parameters of the modalities used in our multimodal imaging pipeline are summarized in Table S1. The imaging principle of each modality is based on an optical interaction between the excitation light and the contrast mechanism in the tissue.
Cardiac optical mapping (COM) is implemented in an individual setup. For COM imaging, samples were superfused with a saline solution gassed with 95%/5% O_2/CO_2 and containing (mM): NaCl, 130; $NaHCO_3$, 24; NH_2PO_4, 1.2; $MgCl_2$, 1; glucose, 5.6; KCl, 4; $CaCl_2$, 1.8, at 37°C and pH 7.4. Samples were mechanically uncoupled by using blebbistatin (10uM) diluted in to the superfusate. Sample loading was performed using the voltage-sensitive dye Di-4-ANEPPS (12 μl of a stock containing 5 μg/ml DMSO), which was dispersed directly over the tissue slice and allowed to incubate for 1 minute. The signals were detected through a 650 ± 20 nm band-pass filter with 100 × 100 pixels at 1 kHz and a spatial resolution of 0.3 mm × 0.3 mm after 530 nm excitation [2]. Pacing was applied to the cardiac sample at two fixed frequencies, 3 Hz and 1.5 Hz.
Optical coherence tomography (OCT), multiphoton microscopy (MPM) including second harmonic generation (SHG) and two-photon excitation fluorescence (TPEF) and line scan Raman microspectroscopy (LSRM) are in a multimodal optical imaging setup with a common sample path [4–6]. The switching between the modalities was performed with flip mirrors. A galvanometric mirror pair was used for scanning. OCT operated in a low- and high-magnification mode, why two lateral resolution values are listed. A low numerical aperture (NA) objective (4x, CFI E Plan Achromat Objective, 0.1 NA, Nikon, Japan) was used for the low-magnification OCT mode facilitating easy intermodal correlation to high-magnification OCT mode using a high NA objective (16x, CFI LWD Plan Fluorite Objective, 0.8 NA). In low-magnification OCT mode 10 sub-volumes were acquired and stitched together with a minimum overlap of about 500 μm to obtain a FOV of 5.3 mm × 4.4 mm. For easy and fast image stitching, a 3D translation stage was used and translated with discrete steps across a well-defined coordination grid. For two-dimensional (2D) representation, maximum intensity projections were performed across the entire sample thickness of about 400 μm. The high-magnification OCT mode operated on a reduced FOV of 560 μm × 560 μm but with an increased lateral resolution of 1 μm at a similar axial resolution. The slightly different FOV for MPM covering 506 μm × 506 μm was caused by the non-uniformity of illumination. At each ROI, multimodal images were acquired starting from the surface and then moving up to 250 μm into the tissue with 5 μm interval steps.
The OCT software enabled real-time previews at a rate of 20 B-scans per second, which is essential for 3D navigation and screening across the sample and intermodal correlation without any discernible time lag. SHG and TPEF images were acquired in two channels simultaneously with 512 × 512 pixels and a frame rate of 1.2 frames/s and 20 times averaging using the free software ScanImage to control the scanning and the detection. Analysis and further postprocessing were done with custom-written MATLAB scripts.
For multimodal imaging, slices were fixed with two metal clips on modeling clay to isolate the cardiac tissue from underlying substrates. The metal clips were put at positions close to the slice borders to avoid tissue damage. The mounted tissue was put in a petri dish and flooded with distilled water to decrease the difference in refractive index between tissue and environment.

Table S1: System Parameters of the multimodal optical imaging pipeline

Modality	Excitation	Detection	FOV	Resolution	Contrast Mechanism
COM	530 nm	630 − 670 nm	18 mm × 12 mm	lateral: 0.7 mm	Fluorescence of potentiometric dyes
OCT	700 − 900 nm	700 − 900 nm	2.25 mm × 2.25 mm<br>560 μm × 560 μm	lateral: 4 μm<br>lateral: 1 μm<br>axial: 2.2 μm	Changes in index of refraction
SHG	800 nm	400 nm	506 μm × 506 μm	lateral: 1 μm<br>axial: 2.7 μm	Non-Centrosymmetric structures
TPEF		550 nm			Autofluorescence of NADH/FAD
LSRM	785 nm	825 − 1020 nm	80 μm × 250 μm	lateral: 10 μm<br>spectral: 0.5 nm	Molecular vibrational modes

Raman spectra were collected across a line within 10 minutes performing 51 stops to increase the acquisition speed compared to standard point scanning RS [7]. Each Raman image was acquired in 10 sec and accumulated once. Processing of the Raman spectra followed the protocol established by *Bocklitz et al*[8]. In brief, fluorescent background was removed, and smoothing was applied by Savitzky-Golay filtering [9, 10]. Outlier removal and data standardization was performed on all processed 459 spectra. An open-source PLS-DA MATLAB toolbox was used for multiclass classification [11]. The parameters used in the PLS-DA toolbox were as follows: The samples were labeled according to the histopathologic classification. Centering and scaling were applied. Hard PLS-DA with 12 PLS-DA components was chosen. Type I error was set to 0.05, and the outlier level was set to 0.01.

III Radiomic Analysis

III.1 Image Preparation

For radiomic analysis, binning was performed to change image size for each MPM modality from 512×512 pixels to 128×128 pixels, as shown in Figure S1a, S1b and S1e. The corresponding co-registered OCT image in en face is shown in Figure S1d. A mask was automatically generated for each modality using Fiji [12] (s. Figure S1c). In case of manual masking, background noise in each SHG image was removed manually by applying a threshold. An threshed SHG image is shown in Figure S1f. Signal renormalization was performed to account for differences in relative intensities of SHG and TPEF. The intensity values of each image were divided by the average value of the background and the generated renormalized images were binned down to 8 bits using a linear rescaling. 128×128 pixels 8-bit images were obtained for both modalities and one binary mask. These features demonstrated 'strong' to 'very strong' reproducibility across multiple centers, as defined by IBSI guidelines [13–15]. Monte Carlo cross-validation with 100 stratified folds was conducted where 80% of the samples were randomly allocated to the training set and 20% to the test set. During cross-validation, each fold's preprocessing included the standardization of features, imputation using k-nearest neighbors, selection of features through the minimum redundancy maximum relevance algorithm, addressing data imbalance with the synthetic minority over-sampling technique, and automated hyperparameter tuning via a random search within a pre-defined hyperparameter grid [16, 17].

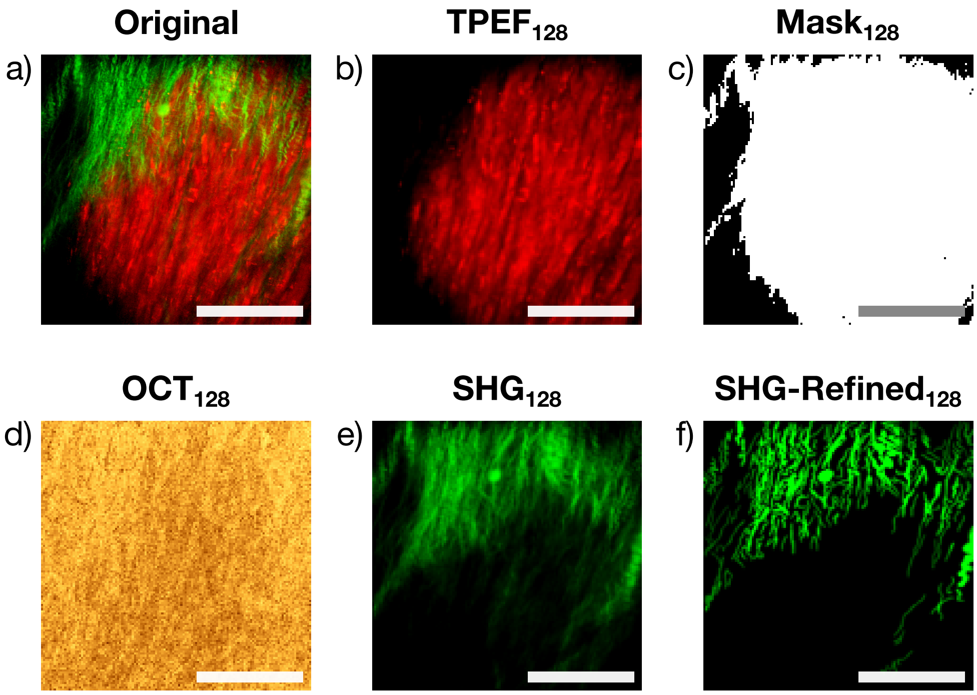

Figure S1: Masking steps used for the machine learning. a) Original 512 × 512 pixels-sized MPM image compromising a SHG channel colored in green and a TPEF channel colored in red. b) and e) are the corresponding isolated TPEF and SHG channels downsized to 128 × 128 pixels. d) Co-registered OCT en face plane downsized from 1600 × 1600 pixels to 128 × 128 pixels. c) Automatically created 128 × 128 pixels mask by thresholding the TPEF image in b) and the SHG image in e) and combine both sub-masks with an OR-condition. The masks is applied for the SHG and TPEF channel. f) Manually thresholding and refinement of the SHG image to remove background noise, which would be otherwise included due to the larger extended mask in c). All scale bars equals 200 μm.

Table S2: Summary of the classifiers used for the cardiac radiomics machine learning analysis. Performance metrics were calculated across all MC folds.

Classifier	ACC	SNS	SPC	PPV	NPV	BACC	AUC
SVM	0.99	1.00	0.98	0.99	1.00	0.99	0.99
LGR	0.99	0.99	0.98	0.99	0.99	0.99	0.99
EBM	1.00	1.00	1.00	1.00	1.00	1.00	1.00
DT	0.99	0.99	1.00	1.00	0.99	1.00	1.00
KNN	1.00	1.00	1.00	1.00	1.00	1.00	1.00
NN	0.98	0.98	0.98	0.99	0.96	0.98	0.98
RF	1.00	1.00	1.00	1.00	1.00	1.00	1.00
XGB	1.00	1.00	1.00	1.00	1.00	1.00	1.00

Table S3: Summary of the classifiers used for the cardiac radiomics machine learning analysis based on manually masked MPM images. Performance values were calculated across all MC folds.

Classifier	ACC	SNS	SPC	PPV	NPV	BACC	AUC
SVM	1.00	1.00	1.00	1.00	1.00	1.00	1.00
LGR	1.00	1.00	1.00	1.00	1.00	1.00	1.00
EBM	0.99	1.00	0.98	0.97	1.00	0.99	0.99
DT	1.00	1.00	1.00	1.00	1.00	1.00	1.00
KNN	0.99	1.00	0.98	0.97	1.00	0.99	0.99
NN	1.00	1.00	1.00	1.00	1.00	1.00	1.00
RF	0.98	1.00	0.97	0.94	1.00	0.98	0.98
XGB	0.97	1.00	0.96	0.93	1.00	0.98	0.98

III.2 Machine Learning Models

The radiomic engine used a mixed random forest ensemble classifier model consisting of eight classifiers [13]. Each MC fold training set was processed based on the classification models SVM (support vector machine), LGR (logistic regression classifier), EBM (explainable boosting machine), DT (decision tree), KNN (k nearest neighbor), NN (neural network), RF (random forest) and XGB (xg boost). Once a classifier model was trained, the corresponding model was evaluated using the validation set of each MC to estimate prediction performance of that model. Across the MC folds, predictive performance was assessed in terms of accuracy (ACC), sensitivity (SNS), specificity (SPC), positive predictive value (PPV), negative predictive value (NPV), balanced accuracy (BACC) and area under the curve (AUC). The performance values for each classifier using automated or manual masked SHG data are summarized in Table S2 or S3, respectively.

References

(1) Dib, N.; Diethrich, E. B.; Campbell, A.; Gahremanpour, A.; McGarry, M.; Opie, S. R. *Journal of Pharmacological and Toxicological Methods* **2006**, *53*, 256–263.

(2) Ramlugun, G. S.; Kulkarni, K.; Pallares-Lupon, N.; Boukens, B. J.; Efimov, I. R.; Bernus, O.; Walton, R. D. *Frontiers in physiology* **2023**, *14*, 734356.

(3) Pallares-Lupon, N.; Bayer, J. D.; Guillot, B.; Caluori, G.; Ramlugun, G. S.; Kulkarni, K.; Loyer, V.; Bloquet, S.; El Hamrani, D.; Naulin, J., et al. *JoVE (Journal of Visualized Experiments)* **2022**, e62909.

(4) Andreana, M.; Le, T.; Hansen, A. K.; Verhoef, A. J.; Jensen, O. B.; Andersen, P. E.; Slezak, P.; Drexler, W.; Fernández, A.; Unterhuber, A. *Journal of Biomedical Optics* **2017**, *22*, 091517.

(5) Andreana, M.; Sentosa, R.; Erkkilä, M. T.; Drexler, W.; Unterhuber, A. *Photochem. Photobiol. Sci.* **2019**, *18*, 997–1008.

(6) Bovenkamp, D.; Sentosa, R.; Rank, E.; Erkkilä, M. T.; Placzek, F.; Püls, J.; Drexler, W.; Leitgeb, R. A.; Garstka, N.; Shariat, S. F., et al. *Applied Sciences* **2018**, *8*, 2371.

(7) Bovenkamp, D.; Micko, A.; Püls, J.; Placzek, F.; Höftberger, R.; Vila, G.; Leitgeb, R.; Drexler, W.; Andreana, M.; Wolfsberger, S.; Unterhuber, A. *Molecules* **2019**, *24*, DOI: `10.3390/molecules24193577`.

(8) Bocklitz, T.; Walter, A.; Hartmann, K.; Rösch, P.; Popp, J. *Analytica Chimica Acta* **2011**, *704*, 47–56.

(9) Lieber, C. A.; Mahadevan-Jansen, A. *Applied Spectroscopy* **2003**, *57*, PMID: 14658149, 1363–1367.

(10) Savitzky, A.; Golay, M. J. E. *Analytical Chemistry* **1964**, *36*, 1627–1639.

(11) Zontov, Y.; Rodionova, O. Y.; Kucheryavskiy, S.; Pomerantsev, A. *Chemometrics and Intelligent Laboratory Systems* **2020**, *203*, 104064.

(12) Schindelin, J.; Arganda-Carreras, I.; Frise, E.; Kaynig, V.; Longair, M.; Pietzsch, T.; Preibisch, S.; Rueden, C.; Saalfeld, S.; Schmid, B., et al. *Nature methods* **2012**, *9*, 676–682.

(13) Papp, L. et al. *European Journal of Nuclear Medicine and Molecular Imaging* **2021**, *48*, 1–11.

(14) Papp, L.; Rausch, I.; Grahovac, M.; Hacker, M.; Beyer, T. *Journal of Nuclear Medicine* **2019**, *60*, 864–872.

(15) Zwanenburg, A. et al. *Radiology* **2020**, *295*, 328–338.

(16) Peng, H.; Long, F.; Ding, C. *IEEE Transactions on pattern analysis and machine intelligence* **2005**, *27*, 1226–1238.

(17) Krajnc, D.; Papp, L.; Nakuz, T. S.; Magometschnigg, H. F.; Grahovac, M.; Spielvogel, C. P.; Ecsedi, B.; Bago-Horvath, Z.; Haug, A.; Karanikas, G., et al. *Cancers* **2021**, *13*, 1249.
